# Proteomic Profiling of Tissue Exosomes Indicates Continuous Release of Malignant Exosomes in Urinary Bladder Cancer Patients, Even with Pathologically Undetectable Tumour

**DOI:** 10.3390/cancers13133242

**Published:** 2021-06-29

**Authors:** Maria Eldh, Michael Mints, Stefanie Hiltbrunner, Sam Ladjevardi, Farhood Alamdari, Markus Johansson, Tomasz Jakubczyk, Rosanne E. Veerman, Ola Winqvist, Amir Sherif, Susanne Gabrielsson

**Affiliations:** 1 Division of Immunology and Allergy, Department of Medicine Solna, Karolinska Institute, 171 64 Stockholm, Sweden; maria.eldh@ki.se (M.E.); michael.mints@ki.se (M.M.); Stefanie.Hiltbrunner@usz.ch (S.H.); rosanne.veerman@ki.se (R.E.V.); 2 Department of Surgical and Perioperative Sciences, Urology and Andrology, Umeå University, 907 36 Umeå, Sweden; amir.sherif@umu.se; 3 Department of Urology, Akademiska University Hospital, 751 85 Uppsala, Sweden; sam.ladjevardi@akademiska.se; 4 Department of Urology, Västmanland Hospital, 721 89 Västerås, Sweden; farhood.alamdari@regionvastmanland.se; 5 Department of Urology, Sundsvall Hospital, Sundsvall, Umeå University, 907 36 Umeå, Sweden; markus.johansson@rvn.se; 6 Department of Urology, Länssjukhuset Ryhov, 553 05 Jönköping, Sweden; tomasz.jakubczyk@rjl.se; 7 Department of Clinical Immunology and Transfusion Medicine, Karolinska University Hospital, 171 76 Solna, Sweden; ola.winqvist@telia.com

**Keywords:** extracellular vesicles, exosomes, urinary bladder neoplasms, neoadjuvant therapy, cystectomy, proteomics

## Abstract

**Simple Summary:**

Urinary bladder cancer (UBC) has a high recurrence rate, and biomarkers for different treatment strategies are highly needed. This study investigated the release of nanovesicles called exosomes from urinary bladder tissue from tumour-proximal sites as well as tumour-distant sites in transurethrally resected (TUR-B) patients with or without preoperative neoadjuvant chemotherapy prior to ensuing radical cystectomy—all without remaining visible tumour after TUR-B. We show that cancer-promoting exosomes were detected from both sites, suggesting that the previous tumour has altered the whole bladder tissue into a cancer-supporting milieu. The exosomes may originate from remaining pathologically undetectable cancer cells or transformed epithelial cells, and the study supports the notion of exosomes as mediators of metastatic spread and as potential biomarkers. It also supports early and radical removal of the bladder in urinary bladder cancer patients.

**Abstract:**

Invasive urothelial bladder cancer (UBC) has high recurrence rates even after radical cystectomy (RC). Exosomes are membrane-bound nanovesicles, which have been shown to contribute to carcinogenesis and metastasis. We previously showed that urinary exosomes display a malignant profile in UBC patients despite the absence of detectable tumour. Here, we investigated exosomes from sampling sites close to or distant from the former tumour, aiming to understand the effect of the tumour on the local milieu. Ten patients scheduled for cystectomy after transurethral bladder resection (TUR-B), without remaining detectable tumour, were included. Exosomes were isolated from tissue explants of both the previous tumour site and distant bladder tissue. Proteins were quantified by mass spectrometry in seven patients. Exosomes from the previous tumour site were enriched in inflammatory but not cancer-related pathways compared to distant tissue. However, the 69 most abundant proteins in tissue-derived exosomes regardless of site, 20 of which were also found in urinary exosomes from our previous study, were enriched for cancer-related metabolic pathways and associated with poor prognosis in an external mRNA dataset. The enrichment of cancer-related pathways in the most abundant proteins, regardless of sampling site, confirms our hypothesis that despite the absence of detectable tumour, the entire bladder releases exosomes that contribute to metastasis and highlights the need for early RC.

## 1. Introduction

Muscle-invasive urothelial bladder cancer (UBC) is a disease with poor prognosis and high recurrence rates [1]. Remaining undetected tumour cells in UBC are supported by the fact that patients with organ-confined disease (pT2N0) after radical cystectomy (RC) still suffer recurrence rates of up to 40% [2]. In addition, it has been shown that survival can be substantially improved by complete downstaging (pT0N0) through transurethral resection of bladder tumour (TUR-B) plus neoadjuvant chemotherapy (NAC) and that patients, completely downstaged only through TUR-B, without receiving NAC, showed significantly poorer overall survival, suggesting the presence of undetected cancer dissemination [3].

Exosomes are small membrane-enclosed extracellular vesicles of endosomal origin, which can act as messengers between cells through horizontal transfer of biomolecules [4]. They are produced by all cells and found in all body fluids, such as plasma and urine [5,6,7,8]. Tumour-derived exosomes play an important role in carcinogenesis, tissue remodelling and metastasis [9,10,11]. Furthermore, they can aid in immune evasion through the induction of apoptosis of immune cells and by stimulating regulatory T-cells [12,13]. Moreover, cell migration is promoted by urine-derived exosomes from high-grade UBC patients [14].

We previously showed in a cohort of completely histopathologically downstaged UBC patients that the exosomes released into the ureter urine differed markedly from those in the bladder urine [15]. The latter were enriched for proteins involved in cancer-related metabolic pathways, suggesting continuous release of exosomes with the ability to cause metabolic re-wiring and pre-metastatic niche establishment in tissue even after complete downstaging [15].

With these facts at hand, the purpose of this study—conducted on the same cohort of completely downstaged patients—was to see whether there are any differences in exosomes from the previous tumour site versus histologically normal distant bladder tissue, possibly finding a source for the carcinogenic urinary exosomes previously identified.

After successful extraction of exosomes from explants from both the tumour site and distant bladder tissue, we performed proteomics and enrichment analysis of the exosomes’ protein content in relation to the site of origin and patient characteristics. The differences found between exosomes from the tumour site and distant tissue mainly consisted in upregulation of inflammatory signalling pathways at the tumour site, with no differences in carcinogenic signalling. However, among the proteins that did not differ between tumour site and distant tissue, the most abundant ones were enriched for carcinogenic metabolic signalling pathways similar to those we previously identified in urinary exosomes, and these were correlated to poor prognosis in an external tumour mRNA dataset. This confirms our hypothesis from the previous study that in UBC patients, even after TUR-B, the release of malignant exosomes takes place in the entire urothelium, not only from the tumour site, which merits early radical cystectomy. In addition, the data strengthen the notion that exosomes are sensitive detectors of transformed cells.

## 2. Materials and Methods

### 2.1. Patients

Ten patients with no tumour remaining in the bladder at radical cystectomy were included. All had previously undergone TUR-B, nine had cT2N0G3 tumours while one was staged cT1N0G3. Six were male (of which three had concomitant prostate cancer) and four female. Seven patients had received NAC before surgery. The two chemo naive patients had, in their respective final post-cystectomy specimens, achieved complete downstaging due solely to the TUR-B-effect. The seven NAC patients in the study, having received both TUR-B plus NAC, were also completely downstaged due to a combined effect of both treatments and were finally classified as complete responders (CR). Urine from the bladder and ureter was taken at the same time as these samples, the data from which has been published [15]. The patient data are found in Table 1. 

At RC, tissue was collected from the TUR-B scar—the site of the original tumour. Non-malignant distant bladder tissue was collected as control. The tumour tissue, from the original tumour site, were then divided and parts were sent for histopathological analysis and staging. All samples were shipped and processed on the day of cystectomy.

### 2.2. Exosome Isolation from Tissue Explants

Tissue explant culture was mainly performed as described by Mincheva-Nilsson et al. [16]. Tissue samples were cut into small pieces (5–6 pieces, approximately 10 mm^3^ each), washed with PBS and incubated for 20 h at 37 °C, 5% CO_2_ in 10 mL AIMV media (Gibco). Exosomes were isolated as previously described [15]. In brief, the supernatant, containing exosomes, was spun at 3000× *g* for 30 min at 4 °C and filtered through a 0.22 µm filter. Exosomes were isolated by ultracentrifugation at 100,000× *g* at 4 °C (Ti45 rotor, Beckman Coulter, Brea, CA, USA) for 2 h, followed by a wash with PBS, and finally resuspended in PBS. The exosomes were stored at −80 °C until further use. Protein concentration was measured by the DC protein assay (Bio-Rad, Hercules, CA, USA).

### 2.3. Flow Cytometry

Bead-based flow cytometry of the exosome-enriched pellet was performed as previously described [15]. Sulphate-aldehyde latex beads (30 µL; 4 μm, 1.3 × 10^9^ beads/mL, Invitrogen, Waltham, MA, USA) were incubated with anti-human CD63 antibody (30 µg; H5C6, BD Pharmingen, Franklin Lakes, NJ, USA) under agitation overnight at RT. The antibody-coated beads were then blocked with 100 mM glycine for 30 min, followed by a wash with 0.5% BSA/PBS. Exosomes were then bound to anti-CD63-coated beads with 1.25 µg exosomes per µL beads (1 µL = 1.3 × 10^5^ beads). The bead–exosome complexes were washed in PBS (10,000× *g*, 10 min), incubated with FITC-conjugated antibodies (dilution 1:100; isotype control mouse IgG1 FITC (MOPC-21, Biolegend, San Diego, CA, USA ), anti-human CD9 FITC (M-L13, BD Pharmingen), anti-human CD63 FITC (H5C6, Biolegend) and anti-human CD81 FITC (5A6, Biolegend) for 30 min at 4 °C, washed in PBS (3000× *g*, 5 min), acquired using a FACS Calibur (BD Bioscience, Franklin Lakes, NJ, USA) and analysed by FlowJo software v7 (TreeStar Inc., Ashland, OR, USA).

### 2.4. Nanoparticle Tracking Analysis

The size and concentration of the exosome-enriched pellet was measured with the Nanosight LM10HSB system (NanoSight, Amesbury, UK). The samples were diluted in PBS and vesicles were measured at ~45 particles/frame, 2 × 108 to 8 × 108 particles/mL, camera level 9 and screen gain of 3. For each sample, five consecutive videos, 60 s each, were recorded in RT while injecting the sample with a syringe pump (speed 50).

### 2.5. Electron Microscopy

A total of 3 µL of the exosome enriched pellet, re-suspended in PBS, from each sample was added to a grid with a glow-discharged carbon-coated supporting film for 3 min. The grid was then rinsed by adding 5 µL distilled water, and the excess water was soaked off by a filter paper. The grid was then stained with 5 µL 1% uranyl acetate in water for 7 s. The samples were examined using a Hitachi HT 7700 electron microscope (Hitachi, Tokyo, Japan) at 80 kV and digital images were taken by a Veleta camera (Olympus, Münster, Germany). 

### 2.6. Proteomics

In total, 14 exosome samples (previous tumour site and distant tissue from 7 patients) were analysed by mass spectrometry. First, proteins were isolated by using a urea-containing buffer and a sonication bath. Total protein concentration was then estimated by the Bradford assay (Bio-Rad, Hercules, CA, USA), with the yields shown in Table S1. Proteins were reduced, alkylated and digested with trypsin. Finally, the samples were purified on Pierce C18 Spin Columns (Thermo Scientific, Waltham, MA, USA), dried and resolved in 0.1% FA to a concentration of 0.3 μg/μL. Peptides were separated in reversed-phase on a C18-column, using a 90 min gradient and electrosprayed onto a Q-Exactive Plus Orbitrap mass spectrometer (ThermoFinnigan, San Jose, CA, USA) Tandem mass spectrometry was performed applying HCD collision-induced dissociation.

The analysed proteins were identified by performing database searches, using the Mascot algorithm embedded in Proteome Discoverer 1.4 (ThermoScientific, San Jose, CA, USA) against proteins from Homo sapiens extracted from UniProtKB database (https://www.uniprot.org/) (January, 2016). In addition, a decoy search database, including common contaminants, and a reverse database, estimated the false discovery rate of identification. The search criteria for identification were set to at least two matching peptides of 95% confidence per protein. A label-free intensity analysis was performed for each sample for quantification.

### 2.7. Data Analysis

All analyses were performed in R 4.0.2. Protein expression values were ln+1-transformed. Principal component analysis (PCA) was run using the FactomineR package [17] with site of origin, gender, concomitant prostate cancer and NAC treatment as qualitative supplementary variables and age and number of NAC cycles as quantitative supplementary variables. T-tests were adjusted for multiple testing with Benjamini–Hochberg correction. Enrichment analyses were performed using the clusterProfiler R package [18] with functional pathways from KEGG [19]. For TCGA analysis, we used the UBC dataset consisting of 412 tumours [20] and examined mRNA expression. All expression values were log2(transcripts-per-million)-transformed before analysis. Only samples with full data on survival and sample purity were kept for analysis, resulting in 399 samples. For each sample, a score was calculated by taking the expression means of all genes in the signature per sample. To account for differences in sample composition, we created a linear model of sample score~sample purity and used the model residuals as the final score. The cutpoint for survival analysis was defined using the maxstat algorithm [21], and survival analysis was performed with the survminer package [22].

## 3. Results

**Figure 1:**
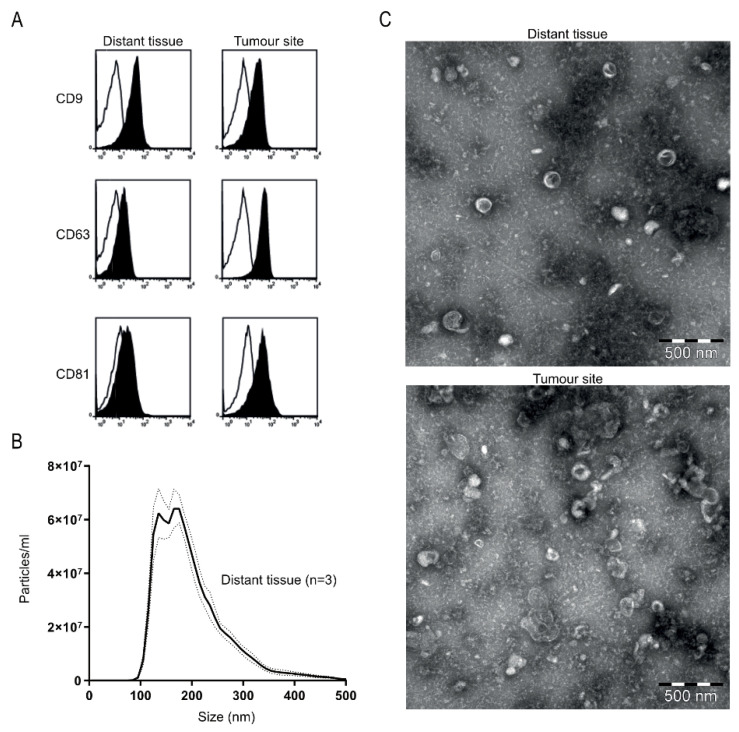
Phenotypic analysis of tissue explant-derived exosomes. (**A**) Exosomes from tissue were bound to anti-human CD63 latex beads, stained for CD9, CD63 and CD81 and analysed by flow cytometry. The histograms are representative of *n* = 2 (black represents marker, line the corresponding isotype control). (**B**) Nanoparticle tracking analysis of exosomes isolated from distant bladder tissue. Data are shown as an average of *n* = 3 and dotted lines indicate ± standard error of the mean. (**C**) Representative transmission electron microscopy images of tissue-derived exosomes isolated from distant bladder tissue (top) and original tumour site (bottom); bar equals 500 nm.

First, we evaluated the phenotype of the tissue-derived extracellular vesicles (EVs) by multiple analyses to determine if they displayed an exosome-like phenotype. Bead-based flow cytometry showed expression of the EV-enriched tetraspanins CD9, CD63 and CD81 (Figure 1A). Furthermore, the EVs showed a typical size distribution and morphology for exosomes, determined by nanoparticle tracking analysis (NTA) and electron microscopy (EM) (Figure 1B,C). In addition, proteomic analysis showed proteins normally enriched in exosomes, such as Rab proteins, annexins, heat shock proteins and tetraspanins (CD9, CD81), and with the ER marker calnexin only detected in some samples and at low levels (Table S2). Together, these data show that the isolated EVs display a predominantly exosomal phenotype with a low contamination of ER-derived cellular debris and are therefore referred to as exosomes.

**Figure 2:**
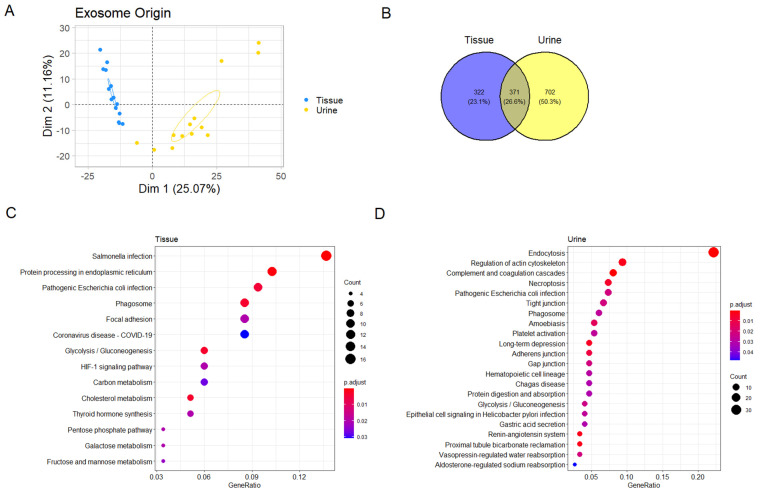
Comparison of tissue- and urine-derived exosomes. (**A**) Principal component analysis (PCA). Ellipses represent 95% confidence intervals. (**B**) Venn diagram of proteins uniquely detected in either urine or tissue. (**C**,**D**) Enrichment plots showing enriched pathways among proteins significantly higher in tissue and urine, respectively.

We then compared the proteomics data from tissue explant-derived exosomes to previously published data on urinary exosomes from the same patients [15]. Principal component analysis (PCA) showed a significant separation based on whether the exosomes were derived from urine or tissue (Figure 2A, Table S3). In total, 693 proteins were identified in tissue-derived exosomes, only 371 of which were also found in urine-derived exosomes (Figure 2B). Taken together, this shows that urine- and tissue-derived exosomes are clearly different (Figure 2B). Furthermore, we performed enrichment analysis on proteins that differed significantly between urine- and tissue-derived exosomes and found large differences in the enriched pathways. Proteins found in exosomes isolated from urine were enriched for many pathways related to cell–cell junctions and electrolyte regulation, while several metabolic pathways were enriched among the proteins only found in the tissue-derived exosomes (Figure 2C,D, Table 2).

**Figure 3:**
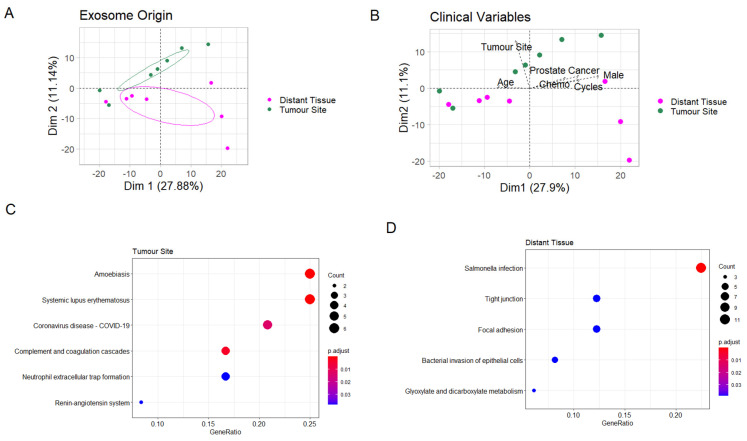
Comparison of exosomes from the tumour site and distant tissue. (**A**) Principal component analysis (PCA). Ellipses represent 95% confidence intervals. (**B**) PCA biplot. The arrows represent clinical parameters, their direction shows how they correlate with the two PCA axes, and their length increases with the correlation value. (**C**,**D**) Enrichment plots showing enriched pathways among proteins significantly higher at the tumour site and in distant tissue, respectively.

The tissue-derived exosomes were thereafter analysed separately. The PCA showed a significant separation between exosomes derived from the tumour site and distant tissue (Figure 3A). This separation was seen in the second dimension (Figure 3A), while the samples separated in the first dimension based on sex, age, chemotherapy treatment and concomitant prostate cancer (Figure 3B), showing that second dimension separation was solely due to the effect of the sampling site and not clinical confounders. A total of 35 proteins correlated significantly with tumour site origin, while 67 correlated with distant tissue in the PCA (Table S4).

No proteins were significantly differentially expressed in the exosomes derived from the tumour site versus distant tissue. The only clinical category except gender that had differentially expressed proteins was exposure to NAC, where CD163 was significantly higher and CD155, or poliovirus receptor (PVR), significantly lower in patients receiving NAC (Figure S1, Table S5).

Thus, in order to analyse the possible functional differences between exosomes derived from tumour site and distant tissue, we performed enrichment analysis on the protein sets correlating significantly with PCA separation in the second dimension. In exosomes derived from the tumour site, the correlating proteins were enriched for immune pathways, representing inflammation through involvement of several complement proteins (Figure 3C), while those correlating with exosomes derived from distant tissue were enriched for two programs related to bacterial infection (Figure 3D). Importantly, no cancer-related pathways were enriched in exosomes derived from the tumour site compared to the distant site (Table 3).

**Figure 4:**
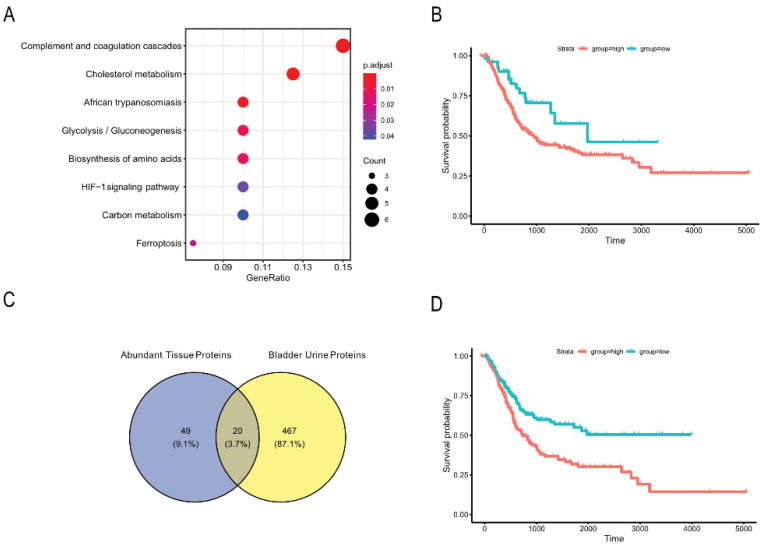
Functional characterisation of the most abundant tissue proteins unrelated to site of origin. (**A**) Enrichment plot showing enriched pathways among proteins that are present in all tissue samples and do not differ significantly by site of origin. (**B**) Kaplan–Meier plot showing the survival of 399 bladder cancer patients [20], separated by their mRNA expression of the 69 most abundant tissue proteins found in this study. (**C**) Venn diagram of proteins enriched in tissue exosomes (distant tissue and original tumour site) and proteins enriched in exosomes from bladder urine compared to ureter urine. (**D**) Kaplan–Meier plot showing the survival of 399 bladder cancer patients [20], separated by their mRNA expression of the 20 proteins upregulated in exosomes derived from bladder vs. ureter urine [15] and being present among the 69 most abundant tissue proteins found in this study.

In order to explore our hypothesis that malignant exosomes are released from all over the bladder, we studied generally abundant proteins in the bladder tissue-derived exosomes, regardless of site. We first excluded all proteins correlating significantly with the second dimension PCA separation, since these proteins were part of the difference between the sampling sites, and then selected only those proteins detected in exosomes from each of the 14 tissue samples, leaving us with 69 proteins, or the ~10% most abundant (Table S6). These proteins represented enrichment of multiple metabolic pathways, including HIF-1 signalling and glycolysis (Figure 4A, Table 3).

We further examined the TCGA UBC dataset [20] for the expression of mRNA transcripts coding for these proteins. The data could be split into two groups based on expression of our protein signature, and patients in the lower expression group had significantly improved survival (*p* = 0.02) compared to the high-expression group (Figure 4B). This suggests that the proteins found in exosomes released from bladder tissue after TUR-B, regardless of tissue site, are not only enriched for potentially cancer-promoting metabolic pathways but are functionally associated with poor prognosis, validating our hypothesis that tumour-promoting exosomes are released across the entire bladder despite the absence of detectable tumour after TUR-B with or without NAC.

The finding that the high expression of these 69 proteins was associated with lower survival made us investigate whether any of these proteins were found in the urine as well. Indeed, 20 of them were also found in urinary exosomes in our previous study [15], where they were derived from urine from the bladder as opposed to the ureters (Figure 4C, Table S7). Importantly, when these 20 proteins were interrogated in the TCGA UBC dataset [20], an even greater effect on long-term survival could be seen (Figure 4C,D).

## 4. Discussion

We previously showed that urinary exosomes from the bladder of completely downstaged patients (pT0N0) contain carcinogenesis-related metabolic proteins [15]. Thus, we now aimed to find out whether, and how, bladder tissue exosomes from these patients differ by site of origin. This could indicate the source of these cancer-linked urinary exosomes.

While extracellular vesicle isolation from tumour tissue explants has been demonstrated in multiple tissue types recently [16,23,24], it has not specifically been implemented for bladder tissue. We here succeeded in isolating tissue-derived exosomes through culturing bladder tissue explants and submitting the culture supernatant to ultracentrifugation. The isolated extracellular vesicles demonstrated the main characteristics of exosomes, showing that the isolation of EVs enriched for exosomes from bladder tissue is feasible.

By comparing proteomic data from exosomes released by the tissue explants with our previously published data on urinary exosomes, we found a clear difference in expressed proteins and enriched pathways between exosomes from tissue and urine. This suggests that tissue and urinary exosomes provide complementary information. As expected from both bladder- and ureter urine-derived exosomes, urine-specific proteins were enriched for pathways involved in electrolyte regulation and cell–cell junctions. Interestingly, for the tissue-derived exosomes, several metabolic pathways were enriched in tissue-specific proteins, suggesting that the release of exosomes that promote recurrence through metabolic rewiring occurs in the entire bladder.

Another factor that could influence the phenotype of released exosomes is NAC, where men are more often treated before surgery compared to women. Furthermore, age showed an inverse relationship with male gender and chemotherapy treatment on PCA. UBC is usually diagnosed later and at a more advanced stage in females compared to men, which could be due to differences in biological effects or diagnostics, as women usually receive treatment for hematuria without further diagnosis [25].

In tissue-derived exosomes, two proteins, CD155 and CD163, were significantly changed in patients who received NAC, while being relatively similar in men versus women.

CD155, or PVR, is involved in many cellular processes, e.g., cell adhesion and tumour immune escape, but it can also result in immune activation by binding its receptor DNAM1 on NK cells; thus, it is of great interest for immunotherapy [26,27,28,29]. PVR has been shown to be overexpressed in many cancers and is associated with poor prognosis. Moreover, soluble PVR levels have been seen to be upregulated in sera of cancer patients, but its role is not yet clear [26,30]. Extracellular vesicles derived from NK cells have been shown to carry DNAM1 and to have cytotoxic activity towards tumour cells [31]. In addition, Zhao et al. showed that pancreas cancer-derived EVs have PVR on their surface [32]. Interestingly, we saw that in tissue-derived exosomes, PVR was downregulated in exosomes by NAC, suggesting that tissue-derived exosomes become less tumour-promoting after NAC. In contrast, the macrophage marker CD163 was upregulated after NAC. Macrophage regulation and balance is critical for cancer outcome [33], and, in bladder cancer, CD163 has been associated with poor prognosis [34]. However, the exact mechanisms, and if exosomes are functional or a shedding mechanism after NAC, remain to be elucidated. In summary, these results confirm the effect of chemotherapy on both cancer cells and immune cells, and their released exosomes, and are interesting in the light of previous studies suggesting that immune activation is necessary for successful chemotherapy [35].

When analysing tissue-derived exosomes alone, we found that there were differences based on the site of origin, with the tumour site being enriched for inflammatory pathways mainly complementing activation. The infection-related pathways (Salmonella/Escherichia coli/COVID-19 infection pathways) likely indicate general inflammation pathways. The proteins in the tissue set that overlap with these pathways are mainly actins, tubulins and ribosomal proteins (Table 2) and are not specific to these infections. In fact, all samples were collected before the COVID-19 pandemic. No differences were found in pathways known to be carcinogenic. This suggests that the differences between exosomes derived from the tumour site and the distant tissue are due to the inflammation caused by TUR-B and not due to remaining microscopic cancer cells at the original tumour site.

Since the above analysis did not suggest a specific origin for the cancer-related metabolic exosomes, we wanted to explore the alternative hypothesis that the entire bladder releases these exosomes evenly and despite no evidence of remaining tumour. We identified a subset of the most abundant proteins in tissue-derived exosomes, that did not correlate with separation by site of origin, and could show that indeed, metabolic pathways similar to the ones previously identified as cancer-related in bladder urine [15] were enriched among these proteins. Further confirming a functional impact, we showed in an external dataset that high expression of these proteins in the tumour is related to poor survival in UBC.

Among the list of the 69 most commonly abundant proteins in tissue-derived exosomes (both distant and original tumour site), which we found to correlate with decreased survival, 20 of these were also found among the proteins enriched in exosomes from bladder urine [15]. That study showed that metabolism-related proteins, enriched in potentially carcinogenic pathways, are specifically upregulated in exosomes from bladder as opposed to ureter urine. Five of these—PGK1, ALDOA, GAPDH, GSTP1 and PKM—are metabolic proteins. This strengthens our hypothesis that exosomes containing proteins involved in metabolic rewiring, found in bladder urine from patients after TUR-B, are released from all across the bladder tissue. Interestingly, analysing just this set of 20 proteins in the TCGA bladder cancer dataset [15] showed that high expression of these proteins is significantly associated with decreased survival (Figure 4D) and should be further investigated as biomarkers in larger cohorts. This is further underlined by the fact that we did not find any of the most commonly described bladder cancer biomarkers, such as NMP22 and BTA [36,37,38], in this study nor in our previous study [15]. Thus, it is likely, and supported by our findings, that other markers are relevant in patients where no tumour is left.

## 5. Conclusions

In conclusion, this study show that it is possible to isolate and analyse exosomes from bladder tissue explants. Importantly, we show that while there are differences in protein expression between exosomes derived from the original tumour site and macroscopically and histopathologically non-tumorous distant bladder tissue, these differences are due to inflammation from scarring and not to a difference in release of malignancy-related exosomes. However, the most abundant proteins in bladder tissue-derived exosomes, regardless of site, are enriched in metabolic carcinogenesis-related pathways and are linked to poor prognosis. This confirms our previous hypothesis that there is a continuous release of malignant exosomes from transformed cells in the entire bladder despite complete downstaging. This is also confirmed by the recurrence and progression rates in patients with muscle invasive bladder cancer who have received both TUR-B and NAC, which is observed especially in patients with stable disease (SD) and progressive disease (PD). Together, this suggests an even distribution of histopathologically undetectable niches of cancerous/precancerous activity in the whole bladder and supports early RC to remove the source of the related bioactive exosomes to reduce the risk of relapse. Our findings also support further development of urinary exosomes as a “liquid biopsy” and as biomarkers for UBC.

## Figures and Tables

**Table 1:** Patient characteristics.

Patient	Preoperative Clinical Stage	Staging Post-Cystectomy	Gender	Age	NAC/noNAC	Number of Cycles	Response	Additional Information
1 ^†#¤^	cT2N0M0,G3 *	pT0N0M0	female	69	NAC	1	CR	
2 ^†¤^	cT2N0M0,G3	pT0N0M0	male	39	NAC	4	CR	
3 ^†^	cT2N0M0,G3	pT0N0M0	male	66	NAC	3	CR	Prostatic cancer Gleason score (3 + 4 = 7)
4 ^†^	cT2N0M0,G3	pT0N0M0	female	79	NAC	3	CR	
5 ^†^	cT2N0M0,G3	pT0N0M0	female	77	noNAC	0	/	
6 ^†^	cT2N0M0,G3	pT0N0M0	male	76	NAC	3	CR	Prostatic cancer Gleason score (3 + 3 = 6)
7 ^†^	cT1N0M0,G3	pT0N0M0	male	57	noNAC	0	/	Prostatic cancer Gleason score (3 + 3 = 6)
8 ^#§^	cT2N0M0,G3	pT0N0M0	male	73	NAC	3	CR	
9 ^§^	cT2N0M0,G2	pT0N0M0	female	67	NAC	3	CR	
10 ^§^	cT2N0M0,G3	pT0N0M0	male	83	noNAC	0	/	

CR = complete response; SD = stable disease; * = in addition to the solid tumour, the patient also had concomitant CIS (cancer in situ); † = mass spectrometry; # = electron microscopy; ¤ = flow cytometry; § = nanoparticle tracking analysis.

**Table 2:** Enriched functional signalling networks in the protein sets differing significantly between urine-derived exosomes (A) and tissue-derived exosomes (B).

A								
ID	Description	Gene Ratio	Bg Ratio	*p* Value	p Adjust	q Value	Gene ID	Count
hsa04144	Endocytosis	33/149	252/8081	1.36 × 10^−19^	3.12 × 10^−17^	2.58 × 10^−17^	VPS37C/CHMP2A/VPS4B/SRC/FOLR1/RAB5A/CAPZA2/RAB7A/IST1/CHMP1B/VPS37D/CHMP4C/MVB12A/CHMP6/TSG101/CHMP4A/EHD4/VPS37B/CHMP1A/VTA1/CHMP5/VPS28/VPS4A/CHMP2B/RAB5C/CDC42/RAB8A/RAB5B/ARF3/RAB35/PDCD6IP/CHMP4B/EHD1	33
hsa04610	Complement and coagulation cascades	12/149	85/8081	4.10 × 10^−8^	4.71 × 10^−6^	3.91 × 10^−6^	MASP2/CFB/SERPINC1/C1QB/C1QC/VWF/SERPIND1/CD55/CR1/F2/CFH/CD59	12
hsa04810	Regulation of actin cytoskeleton	14/149	218/8081	4.62 × 10^−5^	2.36 × 10^−3^	1.95 × 10^−3^	ACTN4/KRAS/EGF/SRC/ITGA3/RDX/IQGAP1/GNG12/BAIAP2/F2/EZR/MSN/CDC42/GNA13	14
hsa04614	Renin–angiotensin system	5/149	23/8081	5.13 × 10^−5^	2.36 × 10^−3^	1.95 × 10^−3^	MME/ACE/ENPEP/ACE2/ANPEP	5
hsa04964	Proximal tubule bicarbonate reclamation	5/149	23/8081	5.13 × 10^−5^	2.36 × 10^−3^	1.95 × 10^−3^	CA2/CA4/AQP1/ATP1A1/MDH1	5
hsa04730	Long-term depression	7/149	60/8081	1.07 × 10^−4^	4.11 × 10^−3^	3.41 × 10^−3^	KRAS/LYN/GNAI3/GNA11/GNAQ/GNAS/GNA13	7
hsa04217	Necroptosis	11/149	159/8081	1.61 × 10^−4^	5.30 × 10^−3^	4.39 × 10^−3^	CHMP2A/VPS4B/CHMP1B/CHMP4C/CHMP6/CHMP4A/CHMP1A/CHMP5/VPS4A/CHMP2B/CHMP4B	11
hsa04520	Adherens junction	7/149	71/8081	3.12 × 10^−4^	8.97 × 10^−3^	7.43 × 10^−3^	ACTN4/YES1/SRC/IQGAP1/PTPRJ/BAIAP2/CDC42	7
hsa05146	Amoebiasis	8/149	102/8081	5.62 × 10^−4^	1.44 × 10^−2^	1.19 × 10^−2^	ACTN4/RAB5A/GNA11/RAB7A/GNAQ/RAB5C/RAB5B/GNAS	8
hsa05130	Pathogenic Escherichia coli infection	11/149	197/8081	1.00 × 10^−3^	2.14 × 10^−2^	1.78 × 10^−2^	SLC9A3R1/MYO1D/SRC/CTTN/ABI1/BAIAP2L1/BAIAP2/F2/EZR/CDC42/GNA13	11
hsa04530	Tight junction	10/149	169/8081	1.10 × 10^−3^	2.14 × 10^−2^	1.78 × 10^−2^	SLC9A3R1/ACTN4/SRC/RDX/CTTN/EZR/MSN/RAB13/CDC42/RAB8A	10
hsa04540	Gap junction	7/149	88/8081	1.15 × 10^−3^	2.14 × 10^−2^	1.78 × 10^−2^	KRAS/EGF/GNAI3/SRC/GNA11/GNAQ/GNAS	7
hsa04962	Vasopressin-regulated water reabsorption	5/149	44/8081	1.21 × 10^−3^	2.14 × 10^−2^	1.78 × 10^−2^	RAB5A/AQP2/RAB5C/RAB5B/GNAS	5
hsa00010	Glycolysis/Gluconeogenesis	6/149	67/8081	1.40 × 10^−3^	2.31 × 10^−2^	1.91 × 10^−2^	ALDOB/GPI/LDHA/LDHB/AKR1A1/TPI1	6
hsa05120	Epithelial cell signalling in Helicobacter pylori infection	6/149	70/8081	1.76 × 10^−3^	2.71 × 10^−2^	2.24 × 10^−2^	ADAM10/LYN/SRC/ATP6V1B1/ATP6V1A/CDC42	6
hsa04145	Phagosome	9/149	152/8081	1.93 × 10^−3^	2.72 × 10^−2^	2.25 × 10^−2^	STX7/LAMP1/LAMP2/ATP6V1B1/RAB5A/RAB7A/ATP6V1A/RAB5C/RAB5B	9
hsa04611	Platelet activation	8/149	124/8081	2.01 × 10^−3^	2.72 × 10^−2^	2.25 × 10^−2^	VWF/LYN/GNAI3/SRC/F2/GNAQ/GNAS/GNA13	8
hsa04640	Hematopoietic cell lineage	7/149	99/8081	2.27 × 10^−3^	2.91 × 10^−2^	2.41 × 10^−2^	CD55/MME/CR1/ITGA3/CD59/ANPEP/CD9	7
hsa04971	Gastric acid secretion	6/149	76/8081	2.69 × 10^−3^	3.10 × 10^−2^	2.57 × 10^−2^	CA2/GNAI3/ATP1A1/EZR/GNAQ/GNAS	6
hsa05142	Chagas disease	7/149	102/8081	2.70 × 10^−3^	3.10 × 10^−2^	2.57 × 10^−2^	C1QB/C1QC/GNAI3/ACE/GNA11/GNAQ/GNAS	7
hsa04974	Protein digestion and absorption	7/149	103/8081	2.85 × 10^−3^	3.12 × 10^−2^	2.58 × 10^−2^	XPNPEP2/SLC3A2/MME/SLC6A19/ACE2/ATP1A1/DPP4	7
hsa04960	Aldosterone-regulated sodium reabsorption	4/149	37/8081	4.57 × 10^−3^	4.78 × 10^−2^	3.96 × 10^−2^	KRAS/SFN/SLC9A3R2/ATP1A1	4
**B**								
**ID**	**Description**	**Gene Ratio**	**Bg Ratio**	***p* Value**	**p Adjust**	**q Value**	**Gene ID**	**Count**
hsa05132	Salmonella infection	16/117	249/8081	5.09 × 10^−7^	1.15 × 10^−4^	1.01 × 10^−4^	HSP90B1/RPS3/MAPK1/ACTR1A/FLNC/TUBA1A/TUBB6/GAPDH/TUBB/CD14/FLNA/ACTB/S100A10/ACTR3/TUBA1B/DYNC1H1	16
hsa04141	Protein processing in endoplasmic reticulum	12/117	171/8081	6.11 × 10^−6^	6.90 × 10^−4^	6.05 × 10^−4^	RPN1/P4HB/HSPA5/HSP90B1/CALR/CANX/ERP29/LMAN1/CKAP4/GANAB/TXNDC5/PDIA3	12
hsa00010	Glycolysis/Gluconeogenesis	7/117	67/8081	4.73 × 10^−5^	3.46 × 10^−3^	3.03 × 10^−3^	ADH1B/PFKM/PFKP/GAPDH/ADH5/PFKL/ALDH9A1	7
hsa04145	Phagosome	10/117	152/8081	6.57 × 10^−5^	3.46 × 10^−3^	3.03 × 10^−3^	CALR/CANX/TUBA1A/TUBB6/MRC2/TUBB/CD14/ACTB/TUBA1B/DYNC1H1	10
hsa04979	Cholesterol metabolism	6/117	50/8081	7.66 × 10^−5^	3.46 × 10^−3^	3.03 × 10^−3^	APOC2/LCAT/LRP1/APOA2/APOC3/APOH	6
hsa05130	Pathogenic Escherichia coli infection	11/117	197/8081	1.24 × 10^−4^	4.68 × 10^−3^	4.10 × 10^−3^	RPS3/MAPK1/MYH11/TMED10/TUBA1A/TUBB6/GAPDH/TUBB/ACTB/ACTR3/TUBA1B	11
hsa04510	Focal adhesion	10/117	201/8081	6.39 × 10^−4^	2.02 × 10^−2^	1.77 × 10^−2^	COL6A1/MAPK1/ILK/FLNC/MYLK/COL6A3/FLNA/ACTB/PPP1CB/TLN1	10
hsa04918	Thyroid hormone synthesis	6/117	75/8081	7.23 × 10^−4^	2.02 × 10^−2^	1.77 × 10^−2^	TTR/SERPINA7/HSPA5/HSP90B1/CANX/ALB	6
hsa00030	Pentose phosphate pathway	4/117	30/8081	8.55 × 10^−4^	2.02 × 10^−2^	1.77 × 10^−2^	PFKM/G6PD/PFKP/PFKL	4
hsa00052	Galactose metabolism	4/117	31/8081	9.71 × 10^−4^	2.02 × 10^−2^	1.77 × 10^−2^	PFKM/PFKP/AKR1B1/PFKL	4
hsa04066	HIF-1 signalling pathway	7/117	109/8081	9.82 × 10^−4^	2.02 × 10^−2^	1.77 × 10^−2^	PFKM/MAPK1/STAT3/PFKP/TF/GAPDH/PFKL	7
hsa00051	Fructose and mannose metabolism	4/117	33/8081	1.23 × 10^−3^	2.33 × 10^−2^	2.04 × 10^−2^	PFKM/PFKP/AKR1B1/PFKL	4
hsa01200	Carbon metabolism	7/117	118/8081	1.56 × 10^−3^	2.72 × 10^−2^	2.38 × 10^−2^	PFKM/G6PD/PFKP/GAPDH/ESD/ADH5/PFKL	7
hsa05171	Coronavirus disease—COVID-19	10/117	232/8081	1.90 × 10^−3^	3.07 × 10^−2^	2.69 × 10^−2^	F13A1/RPSA/RPS2/RPS3/MAPK1/RPL12/STAT3/RPL5/RPL15/RPS4X	10
hsa05230	Central carbon metabolism in cancer	5/117	70/8081	3.34 × 10^−3^	5.03 × 10^−2^	4.41 × 10^−2^	PFKM/G6PD/MAPK1/PFKP/PFKL	5

**Table 3:** Enriched functional signalling networks in the protein sets correlating with previous tumour site (A) and distant tissue (B), respectively, upon principal component analysis (PCA), as well as the most abundant proteins (C) that are present in all tissue-derived exosomal samples and do not differ significantly by site of origin.

A								
ID	Description	Gene Ratio	Bg Ratio	*p* Value	p Adjust	q Value	Gene ID	Count
hsa05146	Amoebiasis	Jun-23	102/8081	2.96 × 10^−^^7^	3.11 × 10^−5^	2.65 × 10^−5^	C9/C8B/CD14/CTSG/RAB5C/GNAS	6
hsa05322	Systemic lupus erythematosus	Jun-23	136/8081	1.62 × 10^−6^	8.52 × 10^−5^	7.26 × 10^−5^	C9/C8B/CTSG/H2AZ1/C6/H3C1	6
hsa04610	Complement and coagulation cascades	Apr-23	85/8081	8.67 × 10^−5^	3.03 × 10^−3^	2.59 × 10^−3^	C9/C8B/F13A1/C6	4
hsa05171	Coronavirus disease—COVID-19	May-23	232/8081	4.12 × 10^−4^	1.08 × 10^−2^	9.21 × 10^−3^	C9/C8B/RPL12/F13A1/C6	5
hsa04614	Renin–angiotensin system	Feb-23	23/8081	1.89 × 10^−3^	3.97 × 10^−2^	3.38 × 10^−2^	CPA3/CTSG	2
**B**								
**ID**	**Description**	**Gene Ratio**	**Bg Ratio**	***p* Value**	**p Adjust**	**q Value**	**Gene ID**	**Count**
hsa05132	Salmonella infection	Nov-49	249/8081	1.97 × 10^−^^7^	3.05 × 10^−5^	2.74 × 10^−5^	DCTN1/RAB5B/MYL9/ARPC2/MAP2K1/ARL8A/DYNLL1/TUBA1C/TUBB2A/ARPC4/CSE1L	11
hsa04530	Tight junction	Jun-49	169/8081	5.08 × 10^−4^	3.91 × 10^−2^	3.50 × 10^−2^	MYL9/HSPA4/ARPC2/RAP1A/TUBA1C/ARPC4	6
hsa00630	Glyoxylate and dicarboxylate metabolism	Mar-49	30/8081	7.58 × 10^−4^	3.91 × 10^−2^	3.50 × 10^−2^	GRHPR/MDH1/SHMT1	3
hsa05100	Bacterial invasion of epithelial cells	Apr-49	77/8081	1.17 × 10^−3^	3.91 × 10^−2^	3.50 × 10^−2^	CAV1/ARPC2/SEPTIN9/ARPC4	4
hsa04510	Focal adhesion	Jun-49	201/8081	1.26 × 10^−3^	3.91 × 10^−2^	3.50 × 10^−2^	COL6A2/MYL9/CAV1/RAP1A/MAP2K1/CAPN2	6
**C**								
**ID**	**Description**	**Gene Ratio**	**Bg Ratio**	***p* Value**	**p Adjust**	**q Value**	**Gene ID**	**Count**
hsa04962	Vasopressin-regulated water reabsorption	Mar-49	44/8081	2.33 × 10^−3^	5.35 × 10^−2^	4.80 × 10^−2^	DCTN1/RAB5B/DYNLL1	3
hsa01240	Biosynthesis of cofactors	May-49	156/8081	2.42 × 10^−3^	5.35 × 10^−2^	4.80 × 10^−2^	NAPRT/AKR1A1/SHMT1/SPR/MTHFD1	5
hsa04610	Complement and coagulation cascades	Jun-40	85/8081	3.27 × 10^−6^	2.64 × 10^−4^	2.37 × 10^−4^	SERPINA1/A2M/KNG1/VTN/SERPING1/CLU	6
hsa04979	Cholesterol metabolism	May-40	50/8081	4.13 × 10^−6^	2.64 × 10^−4^	2.37 × 10^−4^	APOA1/APOE/APOA2/APOC3/APOH	5
hsa05143	African trypanosomiasis	Apr-40	37/8081	3.02 × 10^−5^	1.29 × 10^−3^	1.16 × 10^−3^	KNG1/APOA1/HBB/HBA1	4
hsa00010	Glycolysis/Gluconeogenesis	Apr-40	67/8081	3.15 × 10^−4^	1.01 × 10^−2^	9.04 × 10^−3^	PGK1/ALDOA/GAPDH/PKM	4
hsa01230	Biosynthesis of amino acids	Apr-40	75/8081	4.86 × 10^−4^	1.24 × 10^−2^	1.11 × 10^−2^	PGK1/ALDOA/GAPDH/PKM	4
hsa04216	Ferroptosis	Mar-40	41/8081	1.05 × 10^−3^	2.24 × 10^−2^	2.01 × 10^−2^	CP/TF/FTH1	3
hsa04066	HIF-1 signalling pathway	Apr-40	109/8081	1.97 × 10^−3^	3.60 × 10^−2^	3.23 × 10^−2^	PGK1/TF/ALDOA/GAPDH	4
hsa01200	Carbon metabolism	Apr-40	118/8081	2.63 × 10^−3^	4.21 × 10^−2^	3.77 × 10^−2^	PGK1/ALDOA/GAPDH/PKM	4

## Data Availability

A publicly available datasets was analysed in this study. It can be found in the TCGA UBC dataset [20]. Additionally, we reanalysed data that we previously published [15]. The data presented in this study are available, deidentified, on request from the corresponding author. The data are not publicly available due to privacy concerns.

## Supplementary Materials

The following are available online at https://www.mdpi.com/article/10.3390/cancers13133242/s1, Figure S1: Expression of CD163 and PVR that differed significantly in patients receiving chemotherapy. Each dot represents the average expression of both distant tissue and tumour site per patient, Table S1: Exosomal yield (mg), Table S2: List of all identified proteins with log-fold change in average expression and p-value (derived from Benjamini-Hochberg-corrected t-test) between tissue and urine, Table S3: Proteins and sample categories significantly correlating with the PCA performed on urine and tissue samples, Table S4: Proteins and sample categories significantly correlating with PCA performed on tissue samples only, Table S5: List of all identified proteins with log-fold change in average expression and adjusted p-values comparing samples based on site of origin, gender, neoadjuvant chemotherapy (NAC) and concomitant prostate cancer, Table S6: List of the most abundant proteins that are present in all tissue samples and do not differ significantly by site of origin, Table S7: 20 proteins upregulated in exosomes derived from bladder vs ureter urine, out of the 69 most abundant tissue proteins.
