# Supplementary figures and images for: Proteomic Profiling of Tissue Exosomes Indicates Continuous Release of Malignant Exosomes in Urinary Bladder Cancer Patients, Even with Pathologically Undetectable Tumour
Source: Cancers (Basel). 2021 Jun 29;13(13):3242. doi: 10.3390/cancers13133242 (PMC8267924; doi:10.3390/cancers13133242)

Protein expression

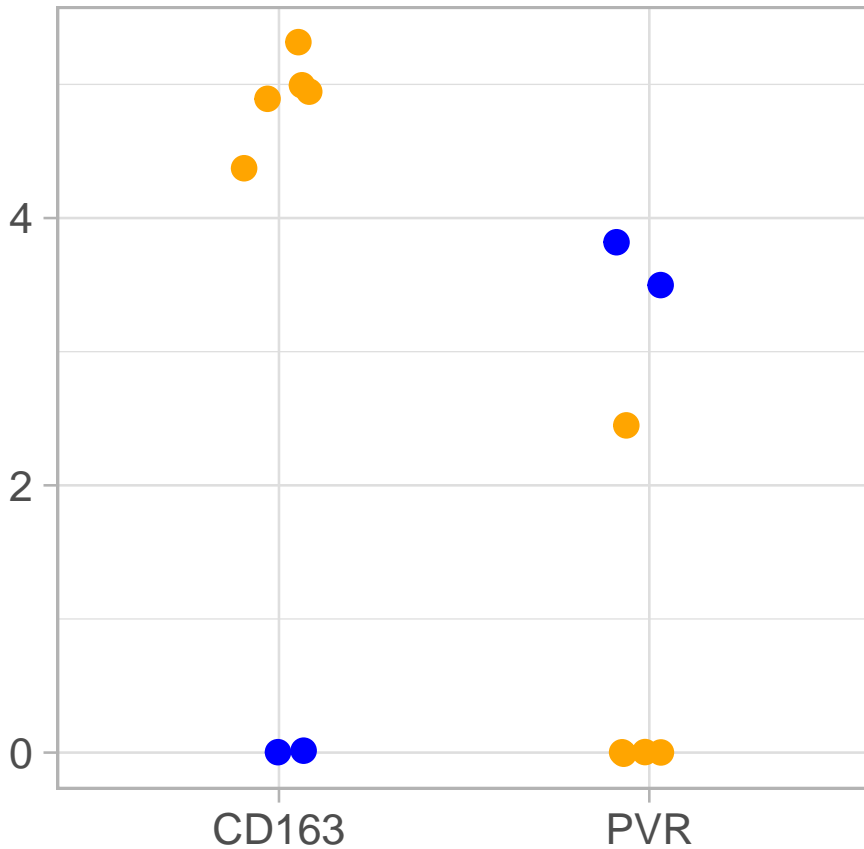

NAC

- Chemo
- No chemo

Supplement: Supplementary file 1 [file cancers-13-03242-s001.zip › Figure S1.pdf]
